# {2,7-Dimeth­oxy-8-[4-(propan-2-yl­oxy)benzo­yl]naphthalen-1-yl}[4-(propan-2-yl­oxy)phen­yl]methanone

**DOI:** 10.1107/S1600536813004959

**Published:** 2013-02-28

**Authors:** Kosuke Sasagawa, Ryo Takeuchi, Taro Kusakabe, Noriyuki Yonezawa, Akiko Okamoto

**Affiliations:** a Department of Organic and Polymer Materials Chemistry, Tokyo University of Agriculture & Technology, Koganei, Tokyo 184-8588, Japan

## Abstract

The title compound, C_32_H_32_O_6_, crystallized with two independent molecules in the asymmetric unit. Each molecule has essentially the same feature of non-coplanar aromatic rings whereby the two 4-isopropoxybenzoyl groups are twisted in a perpendicular manner to the naphthalene ring and oriented in the same direction (*syn*-orientation). The benzene rings of the aroyl groups make dihedral angles of 16.13 (7) and 25.31 (7)° in the two molecules. These benzene rings make dihedral angles of 88.38 (8) and 75.32 (7)° with the naphthalene ring system in one molecule, and 89.71 (7) and 82.11 (7)° in the other. In the crystal, mol­ecules are linked *via* C—H⋯O hydrogen bonds, forming a three-dimensional network. In one independent molecule, the 2-propyl groups of both isoprop­oxy groups are disordered over two positions with site occupancies of 0.512 (3) and 0.488 (3).

## Related literature

For the synthesis of aroylated naphthalene compounds *via* electrophilic aromatic substitution of naphthalene derivatives, see: Okamoto & Yonezawa (2009 ▶); Okamoto *et al.* (2011 ▶). For structures of closely related compounds, see: Hijikata *et al.* (2010 ▶); Sasagawa *et al.* (2011 ▶, 2012*a* ▶,*b* ▶, 2013 ▶).
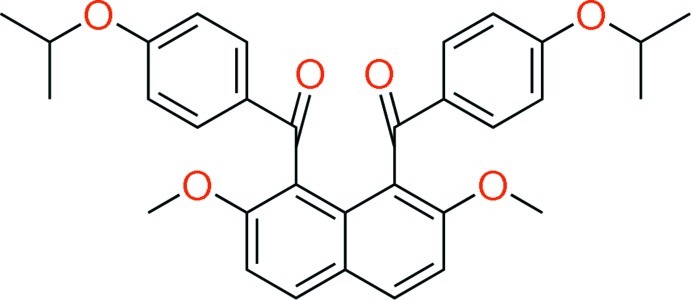


## Experimental

### 

#### Crystal data

C_32_H_32_O_6_
*M* *_r_* = 512.58
Monoclinic, 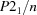

*a* = 10.9988 (2) Å
*b* = 25.8702 (5) Å
*c* = 19.2062 (4) Å
β = 100.338 (1)°
*V* = 5376.27 (17) Å^3^
*Z* = 8
Cu *K*α radiation
μ = 0.70 mm^−1^
*T* = 193 K
0.40 × 0.30 × 0.20 mm

#### Data collection

Rigaku R-AXIS RAPID diffractometer
Absorption correction: numerical (*NUMABS*; Higashi, 1999 ▶) *T*_min_ = 0.766, *T*_max_ = 0.872
98899 measured reflections
9839 independent reflections
8263 reflections with *I* > 2σ(*I*)
*R*_int_ = 0.027

#### Refinement

*R*[*F* ^2^ > 2σ(*F* ^2^)] = 0.041
*wR*(*F* ^2^) = 0.121
*S* = 0.94
9839 reflections
748 parameters
9 restraints
H-atom parameters constrained
Δρ_max_ = 0.27 e Å^−3^
Δρ_min_ = −0.22 e Å^−3^

### 

Data collection: *PROCESS-AUTO* (Rigaku, 1998 ▶); cell refinement: *PROCESS-AUTO*; data reduction: *PROCESS-AUTO*; program(s) used to solve structure: *SHELXS97* (Sheldrick, 2008 ▶); program(s) used to refine structure: *SHELXL97* (Sheldrick, 2008 ▶); molecular graphics: *ORTEPIII* (Burnett & Johnson, 1996 ▶); software used to prepare material for publication: *SHELXL97*.

## Supplementary Material

Crystal structure: contains datablock(s) I, global. DOI: 10.1107/S1600536813004959/su2564sup1.cif

Structure factors: contains datablock(s) I. DOI: 10.1107/S1600536813004959/su2564Isup2.hkl

Supplementary material file. DOI: 10.1107/S1600536813004959/su2564Isup3.cml

Additional supplementary materials:  crystallographic information; 3D view; checkCIF report

## Figures and Tables

**Table 1:** Hydrogen-bond geometry (Å, °)

*D*—H⋯*A*	*D*—H	H⋯*A*	*D*⋯*A*	*D*—H⋯*A*
C4—H4⋯O7^i^	0.95	2.44	3.3240 (19)	155
C38—H38⋯O2^ii^	0.95	2.52	3.3888 (19)	152
C62—H62⋯O1^iii^	1.00	2.51	3.238 (2)	129

Symmetry codes: (i) 
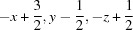
; (ii) 
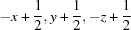
; (iii) 
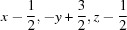
.

**Fig. 1.**
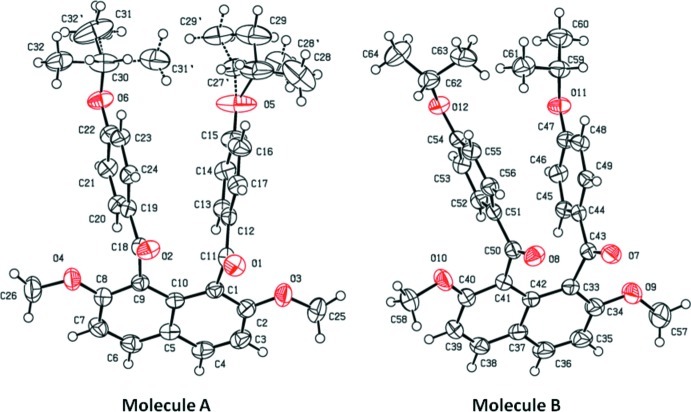
A view of the molecular structure of the two independent molecules, A and B, of the title compound, with atom labelling. Displacement ellipsoids are drawn at the 50% probability level.

**Fig. 2.**
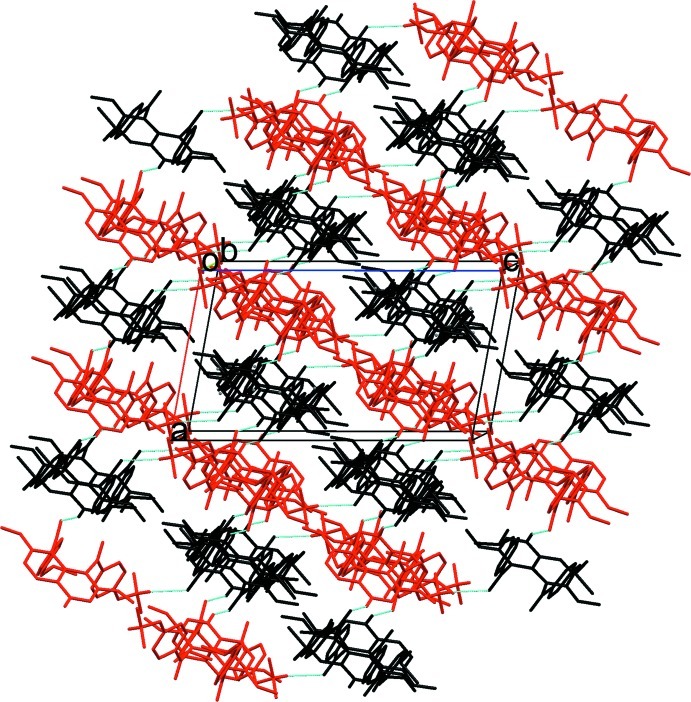
A view along the b axis of the crystal packing title compound, showing the C—H···O hydrogen bonds as dashed lines [black molecule A; red molecule B; see Table 1 for details; H atoms not involved in these interactions have been omitted for clarity].

## supplementary crystallographic information

### Comment

In the course of our studies on selective electrophilic aromatic aroylation of the naphthalene ring core, 1,8-diaroylnaphthalene compounds have proved to be formed regioselectively by the aid of a suitable acidic mediator (Okamoto & Yonezawa, 2009, Okamoto *et al.*, 2011). Recently, we have reported the crystal structures of 1,8-diaroylated 2,7-dimethoxynaphthalene derivatives, such as {8-[4-(butoxy)benzoyl]-2,7-dimethoxynaphthalen-1-yl}[4-(butoxy)phenyl]methanone [1,8-bis(4-butoxylbenzoyl)-2,7-dimethoxynaphthalene] (Sasagawa, Muto *et al.*, 2011), [2,7-dimethoxy-8-(4-methoxybenzoyl)-naphthalen-1-yl](4-methoxyphenyl)-methanone chloroform mono solvate [1,8-bis(4-methoxybenzoyl)-2,7-dimethoxynaphthalene] (Sasagawa, Sakamoto *et al.*, 2013), [2,7-dimethoxy-8-(4-propylbenzoyl)-naphthalen-1-yl](4-propylphenyl)-methanone [1,8-bis(4-propylbenzoyl)-2,7-dimethoxynaphthalene] (Sasagawa, Hijikata *et al.*, 2012*a*) and [2,7-dimethoxy-8-{4-(2-methylpropyl)benzoyl}-naphthalen-1-yl]{4-(2-methylpropyl)phenyl}-methanone [1,8-bis(4-isobutylbenzoyl)-2,7-dimethoxynaphthalene] (Sasagawa, Hijikata *et al.*, 2012*b*).

The aroyl groups in these compounds are almost perpendicularly attached to the naphthalene ring system and oriented in opposite directions (*anti*-orientation). Accordingly, to the best of our knowledge, most 1,8-diaroylnaphthalene derivatives have *anti*-oriented structures. Recently, we have also clarified the structure of another 1,8-diaroylnaphthalene derivative in which the two aroyl groups are situated in the same direction (*syn*-orientation), that is, 2,7-dimethoxy-1,8-bis(4-phenoxybenzoyl)naphthalene (Hijikata *et al.*, 2010). As a part of our ongoing studies on the molecular structures of these kinds of homologous molecules, the crystal structure of the title compound, a 1,8-diaroylated naphthalene bearing isopropoxy groups on the aroyl moieties, is reported on herein.

The molecular structure of the title compound is displayed in Fig 1. It crystallizes with two independent molecules, A and B, in the asymmetric unit. In both molecules, the two 4-isopropoxybenzoyl groups are situated in a *syn*-orientation. The dihedral angles between the best planes of the two phenyl rings in molecules A and B are 16.13 (7) and 25.31 (7)°, respectively. The dihedral angles between the best planes of the 4-isopropoxyphenyl rings and the naphthalene ring system are 88.38 (8) and 75.32 (7)° in A, and 89.71 (7) and 82.11 (7)° in B.

The torsion angles between the carbonyl groups and the naphthalene ring system in the molecules A and B [C10—C1—C11—O1 = 96.99 (17)° and C8—C9—C18—O2 = 105.84 (16)° for A; C42—C33—C43—O7 = 106.99 (17)° and C42—C41—C50—O8 = -67.82 (19)° for B] are larger than those between the carbonyl groups and the 4-isopropoxyphenyl rings [O1—C11—C12—C13 = 174.68 (14)° and O2—C18—C19—C20 = -178.60 (14)° for A; O7—C43—C44—C45 = 157.34 (14)° and O8—C50—C51—C52 = 160.32 (14)° for B].

In the crystal, there are C—H···O interactions connecting the molecules so forming of a three-dimensional network (Table 1 and Fig. 2).

### Experimental

The title compound was prepared by S_N_2 reaction of 1,8-bis(4-hydroxybenzoyl)-2,7-dimethoxynaphthalene (1.0 mmol, 428.5 mg), which was obtained *via* S_N_Ar reaction of 1,8-bis(4-fluorobenzoyl)-2,7-dimethoxynaphthalene with sodium hydroxide, with 2-bromopropane (3.0 mmol, 369 mg) and potassium carbonate (2.8 mmol, 387 mg) in *N*,*N*-dimethylformamide (DMF; 2.5 ml). After the reaction mixture was stirred at 333 K for 6 h, it was poured into water (30 ml) and the mixture was extracted with CHCl_3_ (15 ml × 3). The combined extracts were washed with brine. The organic layers thus obtained were dried over anhydrous MgSO_4_. The solvent was removed under reduced pressure to give cake (93% yield). The crude product was purified by recrystallization from methanol (isolated yield 44%; M.p. = 438.8–441.9 K). The isolated product was crystallized from methanol to give colourless plate-like crystals of the title compound, suitable for X-ray diffraction analysis. Spectroscopic data for the title compound is available in the archived CIF.

### Refinement

In molecule A, the propyl moiety of both isopropoxy groups are disordered over two positions with site occupancies of 0.512 (3) and 0.488 (3). Rigid bond restraints were applied to the *U*ij values of the naphthalene ring (C27—O5, C30—C32 and C30—C31') [3 restraints with the DELU command in *SHELXL97*]. Further restraints were used to generate similar *U*ij values for the atoms of the isopropoxy group (6 restraints with the SIMU command in *SHELXL97*). All H atoms were located in a difference Fourier map and were subsequently refined as riding atoms: C—H = 0.95 (aromatic), 0.98 (methyl) and 1.00 (methyne) Å with U_iso_(H) = 1.5U_eq_(C) for methyl H atoms and = 1.2U_eq_(C) for other H atoms.

### Crystal data

**Table:**

C_32_H_32_O_6_	*F*(000) = 2176
*M**_r_* = 512.58	*D*_x_ = 1.267 Mg m^−^^3^
Monoclinic, *P*2_1_/*n*	Cu *K*α radiation, λ = 1.54187 Å
Hall symbol: -P 2yn	Cell parameters from 83451 reflections
*a* = 10.9988 (2) Å	θ = 3.4–68.2°
*b* = 25.8702 (5) Å	µ = 0.70 mm^−^^1^
*c* = 19.2062 (4) Å	*T* = 193 K
β = 100.338 (1)°	Plate, colourless
*V* = 5376.27 (17) Å^3^	0.40 × 0.30 × 0.20 mm
*Z* = 8	

### Data collection

**Table:**

Rigaku R-AXIS RAPID diffractometer	9839 independent reflections
Radiation source: fine-focus sealed tube	8263 reflections with *I* > 2σ(*I*)
Graphite monochromator	*R*_int_ = 0.027
Detector resolution: 10.000 pixels mm^-1^	θ_max_ = 68.2°, θ_min_ = 3.4°
ω scans	*h* = −13→13
Absorption correction: numerical (*NUMABS*; Higashi, 1999)	*k* = −31→31
*T*_min_ = 0.766, *T*_max_ = 0.872	*l* = −23→23
98899 measured reflections	

### Refinement

**Table:**

Refinement on *F*^2^	Secondary atom site location: difference Fourier map
Least-squares matrix: full	Hydrogen site location: inferred from neighbouring sites
*R*[*F*^2^ > 2σ(*F*^2^)] = 0.041	H-atom parameters constrained
*wR*(*F*^2^) = 0.121	*w* = 1/[σ^2^(*F*_o_^2^) + (0.0715*P*)^2^ + 1.6612*P*] where *P* = (*F*_o_^2^ + 2*F*_c_^2^)/3
*S* = 0.94	(Δ/σ)_max_ = 0.001
9839 reflections	Δρ_max_ = 0.27 e Å^−^^3^
748 parameters	Δρ_min_ = −0.22 e Å^−^^3^
9 restraints	Extinction correction: *SHELXL97* (Sheldrick, 2008), Fc^*^=kFc[1+0.001xFc^2^λ^3^/sin(2θ)]^-1/4^
Primary atom site location: structure-invariant direct methods	Extinction coefficient: 0.00071 (6)

### Special details

**Table:**

Experimental. Spectroscopic data for the title compound:^1^H NMR δ (300 MHz, CDCl_3_);0.74(12*H*, s), 3.71(6*H*, s), 4.59(2*H*, m), 6.76(4*H*, br), 7.19(2*H*, d, *J* = 9.3 Hz), 7.63(4*H*, br), 7.92(2*H*, d, *J* = 9.3 Hz) p.p.m.. ^13^C NMR δ (100 MHz, CDCl_3_); 21.80, 30.70, 56.23, 69.56, 111.00, 114.20, 121.54, 125.24, 129.28, 131.32, 131.58, 155.71, 161.46, 194.86 p.p.m.. IR (KBr, cm^-1^); 1663(Ar, naphthalene), 1598(C=O), 1510(Ar, naphthalene). HRMS (*m/z*); [*M* + H]^+^ Calcd for C_32_H_33_O_6_, 513.2285; found, 513.2285
Geometry. Bond distances, angles etc. have been calculated using the rounded fractional coordinates. All su's are estimated from the variances of the (full) variance-covariance matrix. The cell esds are taken into account in the estimation of distances, angles and torsion angles
Refinement. Refinement of *F*^2^ against ALL reflections. The weighted *R*-factor *wR* and goodness of fit *S* are based on *F*^2^, conventional *R*-factors *R* are based on *F*, with *F* set to zero for negative *F*^2^. The threshold expression of *F*^2^ > 2σ(*F*^2^) is used only for calculating *R*-factors(gt) *etc*. and is not relevant to the choice of reflections for refinement. *R*-factors based on *F*^2^ are statistically about twice as large as those based on *F*, and *R*- factors based on ALL data will be even larger.

### Fractional atomic coordinates and isotropic or equivalent isotropic displacement parameters (Å^2^)

**Table:**

	*x*	*y*	*z*	*U*_iso_*/*U*_eq_	Occ. (<1)
O1	0.60693 (10)	0.72303 (4)	0.36326 (6)	0.0497 (4)	
O2	0.48829 (10)	0.74405 (4)	0.21488 (6)	0.0467 (4)	
O3	0.86555 (11)	0.68059 (5)	0.42031 (6)	0.0554 (4)	
O4	0.54422 (11)	0.70364 (4)	0.06137 (5)	0.0512 (4)	
O5	0.90620 (12)	0.92918 (5)	0.32676 (12)	0.0954 (7)	
O6	0.74253 (11)	0.93487 (4)	0.08944 (7)	0.0544 (4)	
C1	0.76067 (13)	0.68814 (5)	0.30413 (7)	0.0361 (4)	
C2	0.84952 (14)	0.66254 (6)	0.35207 (8)	0.0425 (5)	
C3	0.91844 (15)	0.62125 (6)	0.33124 (9)	0.0483 (5)	
C4	0.89840 (15)	0.60671 (6)	0.26233 (9)	0.0475 (5)	
C5	0.81015 (14)	0.63187 (5)	0.21088 (8)	0.0408 (5)	
C6	0.79227 (15)	0.61570 (6)	0.13952 (9)	0.0474 (5)	
C7	0.70616 (16)	0.63802 (6)	0.08864 (9)	0.0484 (5)	
C8	0.63481 (14)	0.67918 (6)	0.10793 (8)	0.0411 (5)	
C9	0.65217 (13)	0.69810 (5)	0.17632 (7)	0.0346 (4)	
C10	0.73873 (13)	0.67378 (5)	0.23110 (7)	0.0348 (4)	
C11	0.69586 (13)	0.73178 (6)	0.33529 (7)	0.0372 (4)	
C12	0.74894 (13)	0.78416 (5)	0.33299 (7)	0.0356 (4)	
C13	0.85671 (13)	0.79273 (6)	0.30592 (8)	0.0389 (4)	
C14	0.90612 (14)	0.84140 (6)	0.30505 (9)	0.0461 (5)	
C15	0.84830 (15)	0.88319 (6)	0.33003 (11)	0.0551 (6)	
C16	0.73928 (15)	0.87583 (6)	0.35594 (10)	0.0521 (5)	
C17	0.69173 (14)	0.82654 (6)	0.35791 (8)	0.0437 (5)	
C18	0.57814 (13)	0.74625 (5)	0.18612 (7)	0.0351 (4)	
C19	0.61980 (12)	0.79548 (5)	0.15887 (7)	0.0338 (4)	
C20	0.72121 (13)	0.79873 (6)	0.12430 (8)	0.0385 (4)	
C21	0.75761 (14)	0.84540 (6)	0.10076 (8)	0.0412 (5)	
C22	0.69634 (14)	0.89068 (6)	0.11305 (8)	0.0408 (5)	
C23	0.59485 (14)	0.88847 (6)	0.14727 (8)	0.0410 (5)	
C24	0.55734 (13)	0.84092 (6)	0.16882 (7)	0.0373 (4)	
C25	0.96293 (16)	0.65966 (8)	0.47107 (9)	0.0566 (6)	
C26	0.5251 (2)	0.69005 (8)	−0.01125 (9)	0.0681 (7)	
C27	0.8623 (6)	0.9742 (2)	0.3725 (6)	0.083 (3)	0.512 (3)
C28	0.8710 (8)	0.9676 (3)	0.4514 (4)	0.118 (3)	0.512 (3)
C29	0.9435 (5)	1.01873 (17)	0.3551 (4)	0.096 (2)	0.512 (3)
C30	0.69254 (19)	0.98401 (7)	0.10301 (13)	0.0672 (7)	
C31	0.7586 (14)	1.0240 (5)	0.0743 (8)	0.106 (5)	0.512 (3)
C32	0.5743 (4)	0.98772 (14)	0.0342 (2)	0.0628 (11)	0.512 (3)
C29'	0.9072 (5)	1.01823 (16)	0.2926 (3)	0.0723 (18)	0.488 (3)
C27'	0.8509 (7)	0.9777 (2)	0.3317 (3)	0.0587 (18)	0.488 (3)
C28'	0.8944 (7)	0.9823 (3)	0.4114 (5)	0.087 (3)	0.488 (3)
C31'	0.7574 (5)	0.98833 (17)	0.1920 (3)	0.0820 (14)	0.488 (3)
C32'	0.7797 (13)	1.0236 (4)	0.0810 (6)	0.061 (2)	0.488 (3)
O7	0.52631 (10)	0.99841 (4)	0.31284 (6)	0.0509 (4)	
O8	0.40037 (10)	1.00696 (4)	0.16112 (6)	0.0510 (4)	
O9	0.45137 (14)	1.04647 (5)	0.43687 (6)	0.0718 (5)	
O10	0.11665 (12)	1.04293 (5)	0.07925 (6)	0.0604 (4)	
O11	0.20901 (10)	0.80048 (4)	0.35548 (6)	0.0469 (3)	
O12	0.05574 (10)	0.81001 (4)	0.08665 (6)	0.0463 (3)	
C33	0.33939 (14)	1.04269 (5)	0.32204 (8)	0.0387 (4)	
C34	0.35879 (17)	1.06694 (6)	0.38724 (8)	0.0502 (5)	
C35	0.28477 (19)	1.10852 (6)	0.40178 (9)	0.0575 (6)	
C36	0.19387 (17)	1.12627 (6)	0.35009 (9)	0.0511 (6)	
C37	0.17281 (14)	1.10447 (5)	0.28170 (8)	0.0412 (4)	
C38	0.08108 (15)	1.12542 (6)	0.22844 (9)	0.0472 (5)	
C39	0.05962 (15)	1.10602 (6)	0.16186 (9)	0.0495 (5)	
C40	0.13021 (15)	1.06377 (6)	0.14563 (8)	0.0439 (5)	
C41	0.22066 (13)	1.04114 (5)	0.19615 (8)	0.0368 (4)	
C42	0.24586 (13)	1.06143 (5)	0.26639 (7)	0.0355 (4)	
C43	0.41773 (13)	0.99499 (5)	0.31764 (7)	0.0374 (4)	
C44	0.35907 (12)	0.94405 (5)	0.32422 (7)	0.0337 (4)	
C45	0.25260 (13)	0.93868 (5)	0.35358 (7)	0.0376 (4)	
C46	0.20605 (13)	0.89039 (6)	0.36397 (8)	0.0401 (5)	
C47	0.26250 (13)	0.84606 (5)	0.34327 (8)	0.0379 (4)	
C48	0.36787 (13)	0.85103 (5)	0.31252 (8)	0.0391 (4)	
C49	0.41534 (13)	0.89952 (5)	0.30440 (7)	0.0365 (4)	
C50	0.29425 (13)	0.99896 (5)	0.16853 (7)	0.0379 (4)	
C51	0.23169 (13)	0.94911 (5)	0.14746 (7)	0.0357 (4)	
C52	0.12774 (13)	0.93272 (6)	0.17363 (7)	0.0386 (4)	
C53	0.07346 (14)	0.88584 (6)	0.15349 (8)	0.0413 (5)	
C54	0.11889 (13)	0.85469 (5)	0.10467 (7)	0.0382 (4)	
C55	0.22100 (14)	0.87104 (6)	0.07710 (8)	0.0419 (5)	
C56	0.27687 (13)	0.91749 (6)	0.09950 (8)	0.0404 (5)	
C57	0.4899 (3)	1.07333 (9)	0.50069 (11)	0.0873 (9)	
C58	0.05196 (17)	1.07207 (7)	0.02149 (9)	0.0539 (6)	
C59	0.24601 (16)	0.75272 (6)	0.32414 (9)	0.0478 (5)	
C60	0.19015 (19)	0.71001 (7)	0.36188 (12)	0.0653 (7)	
C61	0.19847 (18)	0.75223 (7)	0.24587 (10)	0.0592 (6)	
C62	0.08681 (16)	0.77827 (6)	0.02938 (9)	0.0510 (6)	
C63	0.2018 (2)	0.74668 (7)	0.05348 (12)	0.0681 (7)	
C64	−0.0252 (2)	0.74458 (9)	0.00674 (12)	0.0778 (8)	
H3	0.97810	0.60380	0.36500	0.0580*	
H16	0.69790	0.90440	0.37210	0.0630*	
H17	0.61840	0.82150	0.37670	0.0520*	
H20	0.76550	0.76830	0.11700	0.0460*	
H21	0.82490	0.84680	0.07600	0.0490*	
H23	0.55210	0.91910	0.15570	0.0490*	
H24	0.48690	0.83920	0.19100	0.0450*	
H25A	1.04100	0.66250	0.45350	0.0680*	
H25B	0.94580	0.62320	0.47920	0.0680*	
H25C	0.96920	0.67880	0.51560	0.0680*	
H26A	0.50150	0.65350	−0.01670	0.0820*	
H26B	0.60160	0.69570	−0.02970	0.0820*	
H26C	0.45910	0.71150	−0.03760	0.0820*	
H27	0.77450	0.98260	0.35170	0.0990*	0.512 (3)
H28A	0.95810	0.96780	0.47450	0.1770*	0.512 (3)
H28B	0.83310	0.93470	0.46100	0.1770*	0.512 (3)
H28C	0.82760	0.99610	0.47000	0.1770*	0.512 (3)
H29A	0.90920	1.05160	0.36780	0.1440*	0.512 (3)
H29B	0.94600	1.01850	0.30430	0.1440*	0.512 (3)
H29C	1.02750	1.01460	0.38200	0.1440*	0.512 (3)
H30	0.67310	0.98980	0.15130	0.0810*	0.512 (3)
H31A	0.84770	1.01860	0.08930	0.1580*	0.512 (3)
H31B	0.73550	1.05770	0.09140	0.1580*	0.512 (3)
H31C	0.73790	1.02320	0.02250	0.1580*	0.512 (3)
H32A	0.53160	1.02080	0.03580	0.0950*	0.512 (3)
H32B	0.51640	0.95930	0.03690	0.0950*	0.512 (3)
H32C	0.60610	0.98520	−0.01020	0.0950*	0.512 (3)
H4	0.94490	0.57890	0.24820	0.0570*	
H6	0.84180	0.58850	0.12670	0.0570*	
H7	0.69430	0.62610	0.04110	0.0580*	
H13	0.89650	0.76450	0.28780	0.0470*	
H14	0.98040	0.84640	0.28720	0.0550*	
H27'	0.75880	0.97650	0.31820	0.0710*	0.488 (3)
H28D	0.86570	0.95220	0.43500	0.1290*	0.488 (3)
H28E	0.86040	1.01390	0.42860	0.1290*	0.488 (3)
H28F	0.98480	0.98370	0.42190	0.1290*	0.488 (3)
H29D	0.99740	1.01510	0.30320	0.1090*	0.488 (3)
H29E	0.88290	1.05250	0.30710	0.1090*	0.488 (3)
H29F	0.87820	1.01390	0.24160	0.1090*	0.488 (3)
H30'	0.60150	0.98960	0.08860	0.0810*	0.488 (3)
H31D	0.84700	0.98350	0.19790	0.1240*	0.488 (3)
H31E	0.72210	0.96150	0.21840	0.1240*	0.488 (3)
H31F	0.73980	1.02240	0.21000	0.1240*	0.488 (3)
H32D	0.79360	1.01570	0.03320	0.0920*	0.488 (3)
H32E	0.85860	1.02260	0.11410	0.0920*	0.488 (3)
H32F	0.74320	1.05810	0.08160	0.0920*	0.488 (3)
H35	0.29830	1.12400	0.44740	0.0690*	
H36	0.14320	1.15400	0.36020	0.0610*	
H38	0.03320	1.15380	0.23950	0.0570*	
H39	−0.00230	1.12070	0.12660	0.0590*	
H45	0.21180	0.96860	0.36650	0.0450*	
H46	0.13500	0.88730	0.38540	0.0480*	
H48	0.40640	0.82130	0.29740	0.0470*	
H49	0.48840	0.90260	0.28480	0.0440*	
H52	0.09410	0.95410	0.20560	0.0460*	
H53	0.00450	0.87460	0.17300	0.0500*	
H55	0.25210	0.85050	0.04320	0.0500*	
H56	0.34810	0.92810	0.08160	0.0480*	
H57A	0.56480	1.05720	0.52700	0.1050*	
H57B	0.42430	1.07210	0.52910	0.1050*	
H57C	0.50730	1.10940	0.49040	0.1050*	
H58A	0.08780	1.10680	0.02200	0.0650*	
H58B	−0.03530	1.07470	0.02570	0.0650*	
H58C	0.05880	1.05480	−0.02310	0.0650*	
H59	0.33810	0.74960	0.33360	0.0570*	
H60A	0.10030	0.71440	0.35460	0.0780*	
H60B	0.22430	0.71140	0.41260	0.0780*	
H60C	0.20970	0.67650	0.34290	0.0780*	
H61A	0.23360	0.78150	0.22380	0.0710*	
H61B	0.10820	0.75500	0.23700	0.0710*	
H61C	0.22260	0.71980	0.22570	0.0710*	
H62	0.09910	0.80100	−0.01090	0.0610*	
H63A	0.22290	0.72770	0.01310	0.0820*	
H63B	0.18700	0.72210	0.08990	0.0820*	
H63C	0.27020	0.76970	0.07310	0.0820*	
H64A	−0.01170	0.72220	−0.03230	0.0930*	
H64B	−0.09780	0.76640	−0.00890	0.0930*	
H64C	−0.03900	0.72320	0.04670	0.0930*	

### Atomic displacement parameters (Å^2^)

**Table:**

	*U*^11^	*U*^22^	*U*^33^	*U*^12^	*U*^13^	*U*^23^
O1	0.0479 (6)	0.0540 (7)	0.0511 (6)	−0.0050 (5)	0.0194 (5)	−0.0014 (5)
O2	0.0460 (6)	0.0422 (6)	0.0571 (7)	0.0044 (5)	0.0237 (5)	0.0090 (5)
O3	0.0588 (7)	0.0622 (7)	0.0397 (6)	0.0104 (6)	−0.0059 (5)	0.0021 (5)
O4	0.0594 (7)	0.0569 (7)	0.0343 (5)	0.0094 (5)	0.0006 (5)	−0.0043 (5)
O5	0.0495 (8)	0.0346 (6)	0.2053 (19)	0.0011 (5)	0.0312 (10)	−0.0029 (9)
O6	0.0569 (7)	0.0397 (6)	0.0732 (8)	0.0000 (5)	0.0297 (6)	0.0121 (5)
C1	0.0353 (7)	0.0328 (7)	0.0395 (7)	−0.0020 (6)	0.0049 (6)	0.0029 (6)
C2	0.0430 (8)	0.0401 (8)	0.0430 (8)	−0.0006 (6)	0.0039 (7)	0.0040 (6)
C3	0.0433 (9)	0.0426 (8)	0.0565 (10)	0.0061 (7)	0.0020 (7)	0.0092 (7)
C4	0.0451 (9)	0.0353 (8)	0.0623 (10)	0.0073 (6)	0.0102 (7)	0.0020 (7)
C5	0.0420 (8)	0.0318 (7)	0.0496 (9)	0.0004 (6)	0.0109 (7)	−0.0001 (6)
C6	0.0532 (10)	0.0379 (8)	0.0533 (9)	0.0041 (7)	0.0159 (8)	−0.0065 (7)
C7	0.0602 (10)	0.0440 (8)	0.0423 (8)	0.0000 (7)	0.0131 (7)	−0.0095 (7)
C8	0.0453 (8)	0.0405 (8)	0.0374 (8)	−0.0003 (6)	0.0071 (6)	0.0001 (6)
C9	0.0359 (7)	0.0325 (7)	0.0362 (7)	−0.0005 (6)	0.0088 (6)	0.0008 (5)
C10	0.0345 (7)	0.0300 (7)	0.0408 (8)	−0.0021 (5)	0.0091 (6)	0.0014 (5)
C11	0.0357 (8)	0.0443 (8)	0.0307 (7)	0.0011 (6)	0.0035 (6)	0.0015 (6)
C12	0.0353 (7)	0.0400 (7)	0.0301 (7)	0.0025 (6)	0.0020 (5)	−0.0001 (6)
C13	0.0391 (8)	0.0395 (8)	0.0379 (7)	0.0040 (6)	0.0066 (6)	−0.0009 (6)
C14	0.0384 (8)	0.0431 (8)	0.0565 (9)	0.0002 (6)	0.0081 (7)	0.0047 (7)
C15	0.0380 (9)	0.0371 (8)	0.0872 (13)	0.0013 (6)	0.0035 (8)	0.0014 (8)
C16	0.0447 (9)	0.0410 (8)	0.0688 (11)	0.0083 (7)	0.0050 (8)	−0.0094 (8)
C17	0.0378 (8)	0.0464 (8)	0.0470 (8)	0.0056 (6)	0.0079 (6)	−0.0026 (7)
C18	0.0359 (7)	0.0384 (7)	0.0308 (7)	0.0021 (6)	0.0056 (6)	0.0014 (5)
C19	0.0339 (7)	0.0374 (7)	0.0299 (7)	0.0021 (6)	0.0054 (5)	0.0025 (5)
C20	0.0359 (7)	0.0399 (8)	0.0406 (8)	0.0045 (6)	0.0095 (6)	0.0019 (6)
C21	0.0365 (8)	0.0456 (8)	0.0440 (8)	0.0003 (6)	0.0140 (6)	0.0039 (6)
C22	0.0404 (8)	0.0396 (8)	0.0431 (8)	−0.0014 (6)	0.0091 (6)	0.0067 (6)
C23	0.0429 (8)	0.0375 (8)	0.0443 (8)	0.0053 (6)	0.0126 (6)	0.0050 (6)
C24	0.0371 (8)	0.0405 (7)	0.0362 (7)	0.0037 (6)	0.0114 (6)	0.0036 (6)
C25	0.0479 (10)	0.0733 (12)	0.0451 (9)	−0.0020 (8)	−0.0011 (7)	0.0128 (8)
C26	0.0855 (14)	0.0795 (13)	0.0354 (9)	0.0124 (11)	0.0005 (9)	−0.0048 (8)
C27	0.049 (3)	0.039 (3)	0.153 (9)	0.005 (2)	−0.001 (5)	0.001 (4)
C28	0.123 (5)	0.116 (6)	0.121 (6)	−0.031 (4)	0.039 (5)	−0.059 (5)
C29	0.073 (3)	0.046 (2)	0.166 (6)	0.004 (2)	0.011 (4)	−0.015 (3)
C30	0.0615 (11)	0.0407 (9)	0.1052 (14)	−0.0021 (8)	0.0305 (10)	0.0008 (10)
C31	0.093 (7)	0.073 (6)	0.171 (10)	0.023 (4)	0.079 (7)	0.057 (5)
C32	0.071 (2)	0.0461 (19)	0.079 (2)	−0.0026 (17)	0.0342 (12)	0.0125 (17)
C29'	0.077 (3)	0.041 (2)	0.104 (4)	0.0067 (19)	0.030 (3)	0.009 (2)
C27'	0.058 (3)	0.036 (2)	0.083 (4)	0.0097 (17)	0.015 (3)	0.008 (2)
C28'	0.092 (5)	0.071 (4)	0.098 (6)	−0.008 (4)	0.021 (4)	−0.015 (4)
C31'	0.082 (3)	0.051 (2)	0.112 (2)	0.002 (2)	0.015 (2)	−0.009 (2)
C32'	0.078 (5)	0.029 (3)	0.079 (4)	−0.008 (3)	0.020 (3)	0.000 (3)
O7	0.0382 (6)	0.0428 (6)	0.0710 (8)	−0.0034 (5)	0.0078 (5)	0.0089 (5)
O8	0.0403 (6)	0.0504 (6)	0.0635 (7)	−0.0032 (5)	0.0127 (5)	−0.0110 (5)
O9	0.1025 (11)	0.0548 (7)	0.0451 (7)	0.0154 (7)	−0.0220 (7)	−0.0060 (6)
O10	0.0772 (8)	0.0568 (7)	0.0392 (6)	0.0241 (6)	−0.0108 (6)	−0.0028 (5)
O11	0.0514 (6)	0.0354 (5)	0.0593 (7)	−0.0050 (5)	0.0244 (5)	0.0011 (5)
O12	0.0514 (6)	0.0398 (6)	0.0488 (6)	−0.0040 (5)	0.0121 (5)	−0.0071 (5)
C33	0.0445 (8)	0.0305 (7)	0.0394 (8)	−0.0023 (6)	0.0031 (6)	0.0017 (6)
C34	0.0676 (11)	0.0377 (8)	0.0404 (8)	0.0019 (7)	−0.0032 (8)	0.0002 (6)
C35	0.0861 (13)	0.0402 (9)	0.0434 (9)	0.0035 (8)	0.0045 (9)	−0.0096 (7)
C36	0.0649 (11)	0.0349 (8)	0.0541 (10)	0.0031 (7)	0.0126 (8)	−0.0081 (7)
C37	0.0442 (8)	0.0310 (7)	0.0481 (8)	−0.0004 (6)	0.0079 (7)	−0.0030 (6)
C38	0.0440 (9)	0.0344 (7)	0.0620 (10)	0.0076 (6)	0.0062 (7)	−0.0033 (7)
C39	0.0454 (9)	0.0422 (8)	0.0558 (10)	0.0097 (7)	−0.0044 (7)	0.0003 (7)
C40	0.0456 (9)	0.0402 (8)	0.0428 (8)	0.0055 (6)	−0.0006 (7)	−0.0020 (6)
C41	0.0370 (8)	0.0324 (7)	0.0396 (8)	0.0015 (6)	0.0031 (6)	0.0000 (6)
C42	0.0373 (7)	0.0290 (7)	0.0399 (8)	−0.0016 (6)	0.0058 (6)	−0.0001 (5)
C43	0.0382 (8)	0.0353 (7)	0.0364 (7)	−0.0007 (6)	0.0002 (6)	0.0035 (6)
C44	0.0333 (7)	0.0341 (7)	0.0319 (7)	0.0009 (5)	0.0009 (5)	0.0025 (5)
C45	0.0373 (8)	0.0363 (7)	0.0388 (7)	0.0035 (6)	0.0056 (6)	−0.0025 (6)
C46	0.0365 (8)	0.0422 (8)	0.0438 (8)	−0.0011 (6)	0.0131 (6)	−0.0021 (6)
C47	0.0396 (8)	0.0350 (7)	0.0397 (7)	−0.0031 (6)	0.0084 (6)	0.0028 (6)
C48	0.0389 (8)	0.0342 (7)	0.0457 (8)	0.0032 (6)	0.0114 (6)	0.0021 (6)
C49	0.0335 (7)	0.0373 (7)	0.0394 (7)	0.0013 (6)	0.0084 (6)	0.0035 (6)
C50	0.0388 (8)	0.0388 (7)	0.0346 (7)	0.0045 (6)	0.0025 (6)	0.0006 (6)
C51	0.0350 (7)	0.0356 (7)	0.0346 (7)	0.0074 (6)	0.0009 (6)	0.0010 (6)
C52	0.0397 (8)	0.0421 (8)	0.0338 (7)	0.0042 (6)	0.0060 (6)	−0.0027 (6)
C53	0.0410 (8)	0.0460 (8)	0.0374 (8)	−0.0011 (6)	0.0083 (6)	−0.0005 (6)
C54	0.0408 (8)	0.0350 (7)	0.0367 (7)	0.0039 (6)	0.0015 (6)	0.0019 (6)
C55	0.0429 (8)	0.0383 (8)	0.0453 (8)	0.0068 (6)	0.0099 (7)	−0.0050 (6)
C56	0.0357 (8)	0.0413 (8)	0.0448 (8)	0.0052 (6)	0.0088 (6)	−0.0005 (6)
C57	0.1187 (19)	0.0798 (15)	0.0492 (11)	0.0174 (13)	−0.0228 (12)	−0.0150 (10)
C58	0.0546 (10)	0.0610 (10)	0.0435 (9)	0.0076 (8)	0.0019 (7)	0.0114 (8)
C59	0.0485 (9)	0.0348 (8)	0.0621 (10)	−0.0002 (6)	0.0155 (8)	0.0028 (7)
C60	0.0669 (12)	0.0440 (9)	0.0878 (14)	−0.0052 (8)	0.0211 (10)	0.0102 (9)
C61	0.0614 (11)	0.0529 (10)	0.0643 (11)	−0.0017 (8)	0.0141 (9)	−0.0089 (8)
C62	0.0618 (11)	0.0448 (9)	0.0470 (9)	−0.0014 (8)	0.0113 (8)	−0.0068 (7)
C63	0.0865 (14)	0.0428 (9)	0.0771 (13)	0.0118 (9)	0.0204 (11)	−0.0031 (9)
C64	0.0810 (15)	0.0766 (14)	0.0776 (14)	−0.0184 (11)	0.0190 (12)	−0.0303 (11)

### Geometric parameters (Å, º)

**Table:**

O1—C11	1.2187 (18)	C29—H29B	0.9800
O2—C18	1.2169 (18)	C29—H29A	0.9800
O3—C2	1.3726 (19)	C29'—H29F	0.9800
O3—C25	1.420 (2)	C29'—H29E	0.9800
O4—C8	1.3686 (19)	C29'—H29D	0.9800
O4—C26	1.417 (2)	C30—H30	1.0000
O5—C15	1.356 (2)	C30—H30'	1.0000
O5—C27	1.585 (8)	C31—H31A	0.9800
O5—C27'	1.405 (6)	C31—H31B	0.9800
O6—C22	1.3614 (19)	C31—H31C	0.9800
O6—C30	1.428 (2)	C31'—H31E	0.9800
O7—C43	1.2174 (18)	C31'—H31F	0.9800
O8—C50	1.2186 (18)	C31'—H31D	0.9800
O9—C34	1.370 (2)	C32—H32A	0.9800
O9—C57	1.407 (2)	C32—H32B	0.9800
O10—C58	1.422 (2)	C32—H32C	0.9800
O10—C40	1.3675 (19)	C32'—H32D	0.9800
O11—C47	1.3569 (17)	C32'—H32E	0.9800
O11—C59	1.4637 (19)	C32'—H32F	0.9800
O12—C54	1.3609 (17)	C33—C34	1.383 (2)
O12—C62	1.461 (2)	C33—C42	1.429 (2)
C1—C2	1.385 (2)	C33—C43	1.5161 (19)
C1—C10	1.4290 (19)	C34—C35	1.407 (2)
C1—C11	1.515 (2)	C35—C36	1.356 (3)
C2—C3	1.409 (2)	C36—C37	1.410 (2)
C3—C4	1.355 (2)	C37—C38	1.410 (2)
C4—C5	1.414 (2)	C37—C42	1.434 (2)
C5—C6	1.413 (2)	C38—C39	1.354 (2)
C5—C10	1.433 (2)	C39—C40	1.408 (2)
C6—C7	1.361 (2)	C40—C41	1.388 (2)
C7—C8	1.411 (2)	C41—C42	1.428 (2)
C8—C9	1.383 (2)	C41—C50	1.5104 (19)
C9—C18	1.5182 (19)	C43—C44	1.4826 (19)
C9—C10	1.4317 (19)	C44—C45	1.395 (2)
C11—C12	1.479 (2)	C44—C49	1.3927 (19)
C12—C13	1.395 (2)	C45—C46	1.378 (2)
C12—C17	1.391 (2)	C46—C47	1.396 (2)
C13—C14	1.373 (2)	C47—C48	1.398 (2)
C14—C15	1.383 (2)	C48—C49	1.3783 (19)
C15—C16	1.391 (2)	C50—C51	1.4834 (19)
C16—C17	1.381 (2)	C51—C52	1.395 (2)
C18—C19	1.4805 (19)	C51—C56	1.389 (2)
C19—C20	1.399 (2)	C52—C53	1.376 (2)
C19—C24	1.392 (2)	C53—C54	1.395 (2)
C20—C21	1.374 (2)	C54—C55	1.391 (2)
C21—C22	1.393 (2)	C55—C56	1.383 (2)
C22—C23	1.395 (2)	C59—C60	1.511 (3)
C23—C24	1.384 (2)	C59—C61	1.500 (3)
C27—C29	1.531 (8)	C62—C63	1.507 (3)
C27—C28	1.511 (14)	C62—C64	1.508 (3)
C27'—C29'	1.488 (8)	C35—H35	0.9500
C27'—C28'	1.524 (11)	C36—H36	0.9500
C30—C31'	1.734 (6)	C38—H38	0.9500
C30—C32'	1.514 (13)	C39—H39	0.9500
C30—C32	1.682 (5)	C45—H45	0.9500
C30—C31	1.430 (14)	C46—H46	0.9500
C3—H3	0.9500	C48—H48	0.9500
C4—H4	0.9500	C49—H49	0.9500
C6—H6	0.9500	C52—H52	0.9500
C7—H7	0.9500	C53—H53	0.9500
C13—H13	0.9500	C55—H55	0.9500
C14—H14	0.9500	C56—H56	0.9500
C16—H16	0.9500	C57—H57A	0.9800
C17—H17	0.9500	C57—H57B	0.9800
C20—H20	0.9500	C57—H57C	0.9800
C21—H21	0.9500	C58—H58A	0.9800
C23—H23	0.9500	C58—H58B	0.9800
C24—H24	0.9500	C58—H58C	0.9800
C25—H25A	0.9800	C59—H59	1.0000
C25—H25B	0.9800	C60—H60A	0.9800
C25—H25C	0.9800	C60—H60B	0.9800
C26—H26B	0.9800	C60—H60C	0.9800
C26—H26C	0.9800	C61—H61A	0.9800
C26—H26A	0.9800	C61—H61B	0.9800
C27—H27	1.0000	C61—H61C	0.9800
C27'—H27'	1.0000	C62—H62	1.0000
C28—H28B	0.9800	C63—H63A	0.9800
C28—H28C	0.9800	C63—H63B	0.9800
C28—H28A	0.9800	C63—H63C	0.9800
C28'—H28D	0.9800	C64—H64A	0.9800
C28'—H28E	0.9800	C64—H64B	0.9800
C28'—H28F	0.9800	C64—H64C	0.9800
C29—H29C	0.9800		
			
C2—O3—C25	118.54 (13)	C30—C31—H31C	110.00
C8—O4—C26	119.37 (13)	H31A—C31—H31B	109.00
C15—O5—C27	115.3 (3)	C30—C31—H31B	109.00
C15—O5—C27'	124.6 (3)	H31A—C31—H31C	109.00
C22—O6—C30	120.53 (14)	C30—C31'—H31E	109.00
C34—O9—C57	119.04 (16)	C30—C31'—H31F	110.00
C40—O10—C58	118.29 (13)	C30—C31'—H31D	109.00
C47—O11—C59	120.09 (12)	H31D—C31'—H31F	109.00
C54—O12—C62	119.41 (12)	H31E—C31'—H31F	109.00
C2—C1—C11	114.82 (12)	H31D—C31'—H31E	109.00
C10—C1—C11	124.96 (12)	C30—C32—H32A	109.00
C2—C1—C10	120.19 (13)	C30—C32—H32B	109.00
O3—C2—C3	123.07 (14)	H32A—C32—H32B	110.00
C1—C2—C3	121.68 (14)	H32A—C32—H32C	109.00
O3—C2—C1	115.25 (13)	H32B—C32—H32C	109.00
C2—C3—C4	119.09 (15)	C30—C32—H32C	109.00
C3—C4—C5	121.72 (15)	H32D—C32'—H32F	110.00
C4—C5—C6	120.03 (14)	H32E—C32'—H32F	109.00
C4—C5—C10	120.01 (13)	H32D—C32'—H32E	109.00
C6—C5—C10	119.96 (13)	C30—C32'—H32D	110.00
C5—C6—C7	121.92 (15)	C30—C32'—H32E	109.00
C6—C7—C8	118.66 (15)	C30—C32'—H32F	109.00
O4—C8—C7	123.46 (14)	C34—C33—C42	119.80 (13)
O4—C8—C9	114.67 (13)	C34—C33—C43	115.02 (13)
C7—C8—C9	121.88 (14)	C42—C33—C43	125.08 (12)
C10—C9—C18	124.76 (12)	O9—C34—C33	115.43 (14)
C8—C9—C10	120.19 (13)	O9—C34—C35	122.80 (14)
C8—C9—C18	115.00 (12)	C33—C34—C35	121.73 (15)
C1—C10—C9	125.45 (12)	C34—C35—C36	119.36 (15)
C5—C10—C9	117.26 (12)	C35—C36—C37	121.52 (15)
C1—C10—C5	117.29 (12)	C36—C37—C38	119.92 (14)
O1—C11—C1	120.51 (13)	C36—C37—C42	119.81 (14)
O1—C11—C12	122.39 (13)	C38—C37—C42	120.27 (13)
C1—C11—C12	117.05 (12)	C37—C38—C39	121.56 (15)
C11—C12—C17	120.42 (13)	C38—C39—C40	119.16 (15)
C13—C12—C17	118.04 (13)	O10—C40—C39	122.71 (14)
C11—C12—C13	121.54 (13)	O10—C40—C41	115.46 (14)
C12—C13—C14	121.00 (14)	C39—C40—C41	121.82 (14)
C13—C14—C15	120.34 (15)	C40—C41—C42	119.91 (13)
O5—C15—C16	125.35 (15)	C40—C41—C50	114.91 (13)
O5—C15—C14	114.88 (15)	C42—C41—C50	124.90 (13)
C14—C15—C16	119.77 (15)	C33—C42—C37	117.70 (12)
C15—C16—C17	119.40 (15)	C33—C42—C41	125.02 (13)
C12—C17—C16	121.42 (14)	C37—C42—C41	117.28 (12)
O2—C18—C19	121.92 (12)	O7—C43—C33	121.34 (12)
C9—C18—C19	117.07 (12)	O7—C43—C44	121.26 (12)
O2—C18—C9	121.01 (12)	C33—C43—C44	117.28 (12)
C18—C19—C20	122.89 (12)	C43—C44—C45	122.19 (12)
C18—C19—C24	119.10 (12)	C43—C44—C49	119.37 (12)
C20—C19—C24	118.00 (13)	C45—C44—C49	118.34 (12)
C19—C20—C21	120.88 (14)	C44—C45—C46	120.63 (13)
C20—C21—C22	120.33 (14)	C45—C46—C47	120.52 (13)
O6—C22—C23	124.72 (14)	O11—C47—C46	115.85 (13)
C21—C22—C23	119.87 (14)	O11—C47—C48	124.82 (12)
O6—C22—C21	115.41 (14)	C46—C47—C48	119.33 (12)
C22—C23—C24	118.95 (14)	C47—C48—C49	119.41 (12)
C19—C24—C23	121.91 (13)	C44—C49—C48	121.73 (13)
C28—C27—C29	111.7 (7)	O8—C50—C41	120.21 (12)
O5—C27—C28	120.1 (5)	O8—C50—C51	121.53 (12)
O5—C27—C29	100.6 (5)	C41—C50—C51	118.19 (12)
C28'—C27'—C29'	111.6 (5)	C50—C51—C52	122.41 (12)
O5—C27'—C29'	111.8 (5)	C50—C51—C56	119.30 (13)
O5—C27'—C28'	94.4 (5)	C52—C51—C56	118.29 (13)
O6—C30—C31'	97.7 (2)	C51—C52—C53	120.59 (13)
O6—C30—C32'	105.5 (5)	C52—C53—C54	120.55 (14)
O6—C30—C32	100.15 (19)	O12—C54—C53	115.69 (13)
C31'—C30—C32'	93.6 (5)	O12—C54—C55	124.87 (13)
C31—C30—C32	91.7 (6)	C53—C54—C55	119.43 (13)
O6—C30—C31	109.6 (6)	C54—C55—C56	119.34 (14)
C2—C3—H3	121.00	C51—C56—C55	121.76 (14)
C4—C3—H3	120.00	O11—C59—C60	104.65 (14)
C5—C4—H4	119.00	O11—C59—C61	110.28 (13)
C3—C4—H4	119.00	C60—C59—C61	112.08 (15)
C5—C6—H6	119.00	O12—C62—C63	111.73 (14)
C7—C6—H6	119.00	O12—C62—C64	104.62 (15)
C8—C7—H7	121.00	C63—C62—C64	111.86 (15)
C6—C7—H7	121.00	C34—C35—H35	120.00
C14—C13—H13	119.00	C36—C35—H35	120.00
C12—C13—H13	120.00	C35—C36—H36	119.00
C13—C14—H14	120.00	C37—C36—H36	119.00
C15—C14—H14	120.00	C37—C38—H38	119.00
C15—C16—H16	120.00	C39—C38—H38	119.00
C17—C16—H16	120.00	C38—C39—H39	120.00
C16—C17—H17	119.00	C40—C39—H39	120.00
C12—C17—H17	119.00	C44—C45—H45	120.00
C19—C20—H20	120.00	C46—C45—H45	120.00
C21—C20—H20	120.00	C45—C46—H46	120.00
C20—C21—H21	120.00	C47—C46—H46	120.00
C22—C21—H21	120.00	C47—C48—H48	120.00
C24—C23—H23	120.00	C49—C48—H48	120.00
C22—C23—H23	121.00	C44—C49—H49	119.00
C23—C24—H24	119.00	C48—C49—H49	119.00
C19—C24—H24	119.00	C51—C52—H52	120.00
O3—C25—H25A	110.00	C53—C52—H52	120.00
O3—C25—H25B	109.00	C52—C53—H53	120.00
O3—C25—H25C	109.00	C54—C53—H53	120.00
H25A—C25—H25B	109.00	C54—C55—H55	120.00
H25A—C25—H25C	110.00	C56—C55—H55	120.00
H25B—C25—H25C	109.00	C51—C56—H56	119.00
O4—C26—H26B	109.00	C55—C56—H56	119.00
O4—C26—H26C	109.00	O9—C57—H57A	109.00
O4—C26—H26A	110.00	O9—C57—H57B	109.00
H26B—C26—H26C	109.00	O9—C57—H57C	110.00
H26A—C26—H26C	109.00	H57A—C57—H57B	110.00
H26A—C26—H26B	109.00	H57A—C57—H57C	109.00
O5—C27—H27	108.00	H57B—C57—H57C	109.00
C29—C27—H27	108.00	O10—C58—H58A	110.00
C28—C27—H27	108.00	O10—C58—H58B	109.00
C29'—C27'—H27'	113.00	O10—C58—H58C	109.00
O5—C27'—H27'	113.00	H58A—C58—H58B	109.00
C28'—C27'—H27'	113.00	H58A—C58—H58C	109.00
H28A—C28—H28B	109.00	H58B—C58—H58C	109.00
C27—C28—H28C	109.00	O11—C59—H59	110.00
C27—C28—H28A	110.00	C60—C59—H59	110.00
C27—C28—H28B	110.00	C61—C59—H59	110.00
H28A—C28—H28C	109.00	C59—C60—H60A	109.00
H28B—C28—H28C	109.00	C59—C60—H60B	109.00
H28D—C28'—H28F	109.00	C59—C60—H60C	109.00
H28E—C28'—H28F	110.00	H60A—C60—H60B	110.00
C27'—C28'—H28D	109.00	H60A—C60—H60C	109.00
C27'—C28'—H28E	109.00	H60B—C60—H60C	109.00
C27'—C28'—H28F	110.00	C59—C61—H61A	109.00
H28D—C28'—H28E	109.00	C59—C61—H61B	109.00
H29A—C29—H29B	110.00	C59—C61—H61C	109.00
H29A—C29—H29C	109.00	H61A—C61—H61B	110.00
C27—C29—H29C	110.00	H61A—C61—H61C	110.00
C27—C29—H29A	109.00	H61B—C61—H61C	109.00
C27—C29—H29B	110.00	O12—C62—H62	110.00
H29B—C29—H29C	109.00	C63—C62—H62	110.00
C27'—C29'—H29D	110.00	C64—C62—H62	109.00
C27'—C29'—H29E	110.00	C62—C63—H63A	109.00
H29D—C29'—H29F	110.00	C62—C63—H63B	109.00
H29E—C29'—H29F	109.00	C62—C63—H63C	109.00
H29D—C29'—H29E	109.00	H63A—C63—H63B	109.00
C27'—C29'—H29F	109.00	H63A—C63—H63C	110.00
C32—C30—H30	117.00	H63B—C63—H63C	110.00
C31—C30—H30	117.00	C62—C64—H64A	110.00
O6—C30—H30	117.00	C62—C64—H64B	110.00
C31'—C30—H30'	119.00	C62—C64—H64C	109.00
C32'—C30—H30'	119.00	H64A—C64—H64B	109.00
O6—C30—H30'	119.00	H64A—C64—H64C	109.00
C30—C31—H31A	109.00	H64B—C64—H64C	109.00
H31B—C31—H31C	109.00		
			
C25—O3—C2—C1	−174.05 (14)	C9—C18—C19—C24	−177.82 (12)
C25—O3—C2—C3	5.4 (2)	C9—C18—C19—C20	1.06 (19)
C26—O4—C8—C7	−4.6 (2)	C18—C19—C20—C21	−179.08 (14)
C26—O4—C8—C9	175.17 (15)	C24—C19—C20—C21	−0.2 (2)
C27—O5—C15—C14	−164.8 (4)	C18—C19—C24—C23	177.24 (13)
C27—O5—C15—C16	15.1 (5)	C20—C19—C24—C23	−1.7 (2)
C15—O5—C27—C28	58.1 (7)	C19—C20—C21—C22	2.1 (2)
C15—O5—C27—C29	−179.0 (4)	C20—C21—C22—C23	−2.2 (2)
C30—O6—C22—C21	−175.94 (16)	C20—C21—C22—O6	177.99 (14)
C30—O6—C22—C23	4.3 (2)	C21—C22—C23—C24	0.4 (2)
C22—O6—C30—C31	178.7 (7)	O6—C22—C23—C24	−179.85 (14)
C22—O6—C30—C32	−85.8 (2)	C22—C23—C24—C19	1.6 (2)
C57—O9—C34—C33	170.37 (18)	C42—C33—C34—O9	−179.32 (14)
C57—O9—C34—C35	−12.0 (3)	C42—C33—C34—C35	3.0 (2)
C58—O10—C40—C39	15.6 (2)	C43—C33—C34—O9	4.1 (2)
C58—O10—C40—C41	−163.06 (14)	C43—C33—C34—C35	−173.55 (15)
C59—O11—C47—C48	−11.9 (2)	C34—C33—C42—C37	−1.7 (2)
C47—O11—C59—C60	167.64 (14)	C34—C33—C42—C41	177.20 (15)
C59—O11—C47—C46	168.50 (13)	C43—C33—C42—C37	174.46 (13)
C47—O11—C59—C61	−71.65 (18)	C43—C33—C42—C41	−6.6 (2)
C62—O12—C54—C53	172.24 (13)	C34—C33—C43—O7	−76.67 (18)
C62—O12—C54—C55	−6.4 (2)	C34—C33—C43—C44	99.37 (16)
C54—O12—C62—C64	−160.28 (14)	C42—C33—C43—O7	106.99 (17)
C54—O12—C62—C63	78.51 (17)	C42—C33—C43—C44	−76.98 (18)
C10—C1—C2—C3	−1.5 (2)	O9—C34—C35—C36	−179.22 (17)
C11—C1—C2—O3	−0.13 (19)	C33—C34—C35—C36	−1.7 (3)
C10—C1—C2—O3	178.01 (13)	C34—C35—C36—C37	−0.9 (3)
C2—C1—C10—C9	−178.23 (14)	C35—C36—C37—C38	−177.19 (16)
C11—C1—C10—C5	179.07 (13)	C35—C36—C37—C42	2.1 (2)
C11—C1—C10—C9	−0.3 (2)	C36—C37—C38—C39	178.93 (16)
C2—C1—C11—O1	−84.97 (17)	C42—C37—C38—C39	−0.3 (2)
C2—C1—C11—C12	92.26 (15)	C36—C37—C42—C33	−0.7 (2)
C11—C1—C2—C3	−179.63 (14)	C36—C37—C42—C41	−179.74 (14)
C2—C1—C10—C5	1.1 (2)	C38—C37—C42—C33	178.52 (14)
C10—C1—C11—C12	−85.78 (17)	C38—C37—C42—C41	−0.5 (2)
C10—C1—C11—O1	96.99 (17)	C37—C38—C39—C40	0.3 (2)
O3—C2—C3—C4	−178.53 (15)	C38—C39—C40—O10	−178.07 (15)
C1—C2—C3—C4	0.9 (2)	C38—C39—C40—C41	0.5 (2)
C2—C3—C4—C5	0.0 (2)	O10—C40—C41—C42	177.33 (13)
C3—C4—C5—C6	179.77 (15)	O10—C40—C41—C50	3.2 (2)
C3—C4—C5—C10	−0.3 (2)	C39—C40—C41—C42	−1.4 (2)
C10—C5—C6—C7	−1.6 (2)	C39—C40—C41—C50	−175.50 (14)
C4—C5—C10—C1	−0.3 (2)	C40—C41—C42—C33	−177.63 (14)
C4—C5—C6—C7	178.35 (16)	C40—C41—C42—C37	1.3 (2)
C4—C5—C10—C9	179.14 (14)	C50—C41—C42—C33	−4.1 (2)
C6—C5—C10—C1	179.69 (14)	C50—C41—C42—C37	174.83 (13)
C6—C5—C10—C9	−0.9 (2)	C40—C41—C50—O8	106.00 (16)
C5—C6—C7—C8	1.3 (2)	C40—C41—C50—C51	−70.86 (17)
C6—C7—C8—C9	1.6 (2)	C42—C41—C50—O8	−67.82 (19)
C6—C7—C8—O4	−178.65 (15)	C42—C41—C50—C51	115.32 (15)
O4—C8—C9—C10	176.07 (13)	O7—C43—C44—C45	157.34 (14)
O4—C8—C9—C18	−6.44 (19)	O7—C43—C44—C49	−18.8 (2)
C7—C8—C9—C10	−4.2 (2)	C33—C43—C44—C45	−18.70 (19)
C7—C8—C9—C18	173.30 (14)	C33—C43—C44—C49	165.13 (12)
C18—C9—C10—C1	5.9 (2)	C43—C44—C45—C46	−174.88 (13)
C18—C9—C10—C5	−173.52 (13)	C49—C44—C45—C46	1.3 (2)
C8—C9—C18—O2	105.84 (16)	C43—C44—C49—C48	176.92 (13)
C8—C9—C10—C1	−176.93 (14)	C45—C44—C49—C48	0.6 (2)
C8—C9—C10—C5	3.7 (2)	C44—C45—C46—C47	−2.0 (2)
C10—C9—C18—C19	103.53 (16)	C45—C46—C47—O11	−179.63 (13)
C8—C9—C18—C19	−73.82 (17)	C45—C46—C47—C48	0.7 (2)
C10—C9—C18—O2	−76.80 (19)	O11—C47—C48—C49	−178.44 (14)
O1—C11—C12—C13	174.68 (14)	C46—C47—C48—C49	1.2 (2)
O1—C11—C12—C17	−5.5 (2)	C47—C48—C49—C44	−1.9 (2)
C1—C11—C12—C13	−2.50 (19)	O8—C50—C51—C52	160.32 (14)
C1—C11—C12—C17	177.35 (13)	O8—C50—C51—C56	−20.2 (2)
C13—C12—C17—C16	0.2 (2)	C41—C50—C51—C52	−22.86 (19)
C11—C12—C13—C14	−179.00 (14)	C41—C50—C51—C56	156.59 (13)
C17—C12—C13—C14	1.2 (2)	C50—C51—C52—C53	−179.08 (13)
C11—C12—C17—C16	−179.66 (15)	C56—C51—C52—C53	1.5 (2)
C12—C13—C14—C15	−1.1 (2)	C50—C51—C56—C55	−178.90 (14)
C13—C14—C15—O5	179.68 (17)	C52—C51—C56—C55	0.6 (2)
C13—C14—C15—C16	−0.2 (3)	C51—C52—C53—C54	−2.2 (2)
C14—C15—C16—C17	1.5 (3)	C52—C53—C54—O12	−177.80 (13)
O5—C15—C16—C17	−178.35 (19)	C52—C53—C54—C55	0.9 (2)
C15—C16—C17—C12	−1.5 (3)	O12—C54—C55—C56	179.67 (13)
O2—C18—C19—C20	−178.60 (14)	C53—C54—C55—C56	1.1 (2)
O2—C18—C19—C24	2.5 (2)	C54—C55—C56—C51	−1.8 (2)

### Hydrogen-bond geometry (Å, º)

**Table:**

*D*—H···*A*	*D*—H	H···*A*	*D*···*A*	*D*—H···*A*
C4—H4···O7^i^	0.95	2.44	3.3240 (19)	155
C38—H38···O2^ii^	0.95	2.52	3.3888 (19)	152
C62—H62···O1^iii^	1.00	2.51	3.238 (2)	129

Symmetry codes: (i) −*x*+3/2, *y*−1/2, −*z*+1/2; (ii) −*x*+1/2, *y*+1/2, −*z*+1/2; (iii) *x*−1/2, −*y*+3/2, *z*−1/2.
